# Effects of whole and steam-flaked corn supplementation on productive performance, serum parameters, and reproductive characteristics of dairy ewes

**DOI:** 10.5194/aab-67-583-2024

**Published:** 2024-12-13

**Authors:** Hande Işıl Akbağ, Cemil Tölü, Türker Savaş, Aynur Konyalı, Baver Coşkun, İsmail Yaman Yurtman

**Affiliations:** 1 Department of Animal Science, Faculty of Agriculture, Çanakkale Onsekiz Mart University, Çanakkale, Türkiye; ☆ retired

## Abstract

This study aimed to determine the effects of whole and steam-flaked corn supplementation on the feed intake, serum parameters, and reproductive performance of dairy ewes. A total of 48 ewes (at the end of their lactation period; 57 ± 1.3 kg body weight, BW; 2.69 ± 0.19 body condition score, BCS) were divided into three treatment groups (16 animals per group): the control group (C), which was fed with alfalfa hay and corn silage; the whole-corn group (WC), which was fed with alfalfa hay, corn silage, and whole corn; and the steam-flaked corn group (FC), which was fed with alfalfa hay, corn silage, and steam-flaked corn. The study was conducted for 15 d before ram introduction and for 30 d during the mating in the breeding season. The WC group had higher dry matter (DM), metabolizable energy (ME), and starch intake values than the FC group ( P<0.0001 ). The BW and BCS values were increased in the groups supplemented with whole corn and steam-flaked corn ( P≤0.05 ). The lambing rate was higher in ewes from the WC and FC groups ( P≤0.05 ). Whole and steam-flaked corn supplementation did not affect the non-return ratio or litter size ( P>0.05 ). The serum glucose concentration was similar among the groups, whereas the serum urea concentration increased with either whole or steam-flaked corn supplementation ( P≤0.05 ). In conclusion, whole and steam-flaked corn supplementation increased the BW, BCS, and lambing rate values in dairy ewes.

## 1 Introduction

Nutrition plays a critical role in animal health, especially with respect to regulating the reproductive performance of small ruminants (Martin et al., 2010; Ding et al., 2024). Flush feeding is a supplementary feeding method that is used during the breeding period to stimulate oestrus synchronization and increase the ovulation and gestation rate in ewes. Moreover, it has been determined that the use of feed rations containing high energy and/or protein contents for flush feeding may increase the ovulation rate in ewes (Koyuncu and Canbolat, 2009; Habibizad et al., 2015; Kebede et al., 2019; Tesfaye et al., 2023). The development of follicles in ewes can be improved by elevating their glucose, leptin, and insulin levels, which are linked to ovarian activity (Boland et al., 2001). Glucose determines the ovulation rate, as glucose uptake increases the blood insulin concentration and, in turn, leads to increased secretion of follicle-stimulating hormone (FSH) and luteinizing hormone (LH) (Landau et al., 1995; Haruna et al., 2009; Wang et al., 2023).

When the feed ration contains no bypass glucose, the glucose production rate in ruminants mainly depends on energy intake and gluconeogenesis (Landau et al., 1992). Ruminants have a limited ability to produce glucose from propionic acid, which is created during the absorption of a feed ration containing carbohydrates from the rumen. Providing glucose as a bypass, which moves digestion away from the rumen to the small intestine, may result in more efficient energy intake for the animal (Rigout et al., 2003; Moran et al., 2014). The main glucose sources in the ruminant diet are wheat, corn, barley, oat, and sorghum grains (Huntington et al., 1997). These grain feeds contain higher starch levels than other feeds, but their starch content, structure, and ruminal degradability characteristics are different (Huntington et al., 1997; Philippeau et al., 1999; Swan et al., 2006). Wheat and corn have the highest starch content (76.0 %), followed by sorghum (71.3 %), barley (64.3 %), and oat (58.1 %) (Gomez et al., 2016). Although the degradability of starch in the rumen varies according to the grain type, the starch of wheat and barley is more degradable in the rumen than sorghum and corn (Van Barneveld, 1999). Not only the type of starch but also the processing technique can affect starch degradability in the rumen; for instance, corn grain is partially degraded in the rumen, whereas steam-flaked corn is highly degraded in the rumen (Landau et al., 1992, 1995). It has also been determined that steam flaking increased the level of fermented starch in the rumen compared with extruded corn (Palizdar et al., 2014). Flaking was the most effective method to increase intraruminal degradability in corn processed using different processing techniques, such as extruded, steam-flaked, and rolled and roasted corn, according to a study conducted by Karami et al. (2018). However, there is limited existing research in the literature evaluating the effects of different processing techniques on the reproductive performance of ewes (Landau et al., 1992, 1995).

Therefore, this study hypothesized that supplementary feeding of whole corn to ewes during the breeding season would increase the lambing ratio by increasing the ovulation rate, as it would provide more glucose support to the small intestine compared with steam-flaked corn. The present study aimed to evaluate the effect of whole and steam-flaked corn supplementation on performance, some serum parameters, and the reproductive characteristics of dairy ewes.

## 2 Materials and methods

The experiment was carried out at the Animal Production Research, Production and Practise Unit of the Faculty of Agriculture at Çanakkale Onsekiz Mart University in Çanakkale, northwestern Türkiye (40°07^′^38.82^′′^ N, 26°26^′^24.66^′′^ E; 38 m altitude).

### 2.1 Animals and diets

During the breeding season (August–September 2017), 48 Tahirova (East Friesian × Kıvırcık) ewes (57 ± 1.3 kg body weight, BW; 2.69 ± 0.19 body condition score, BCS; 1–4 years of age) at the end of their lactational period were housed in group barns with wheat straw bedding and free access to water. These animals were randomly allocated into three experimental groups for this work.

The alfalfa hay and corn silage used as roughage sources in the experiment were purchased from Rasyonel Agriculture (Ind., Kırklareli, Türkiye), while whole corn and flaked corn were supplied from Atılım Feed Factory (Bursa, Türkiye). In the study, an experimental ration was offered for 15 d before the ram was released into the groups and for 30 d during mating, followed by a 5 d adaptation period to the experimental diet. The ewes were divided into three groups according to their nutrition type: the first group was the control (C, n=16 ), which was only fed with roughage sources (alfalfa hay and corn silage); the second group was the whole-corn group, which was fed with whole corn in addition to roughage sources (WC, n=16 ); and the third group was the steam-flaked corn group (FC, n=16 ), which was fed with steam-flaked corn as well as roughage sources. The daily ration of ewes in the C group was calculated to be 1.2 times higher than the recommended energy requirements of a 60 kg adult ewe for the breeding season (2.21 ME Mcal d^−1^, where ME denotes metabolizable energy), as per NRC (2007). The WC and FC groups' rations were calculated so that they had an ME content that was 1.5 times higher than the C group's ration.

In the experiment, the feed was divided into two equal meals that were offered at 09:30 LT (local time) and at 17:30 LT, respectively. The roughage sources (alfalfa hay and corn silage) were given to the ewes in a group setting, whereas the grain sources (whole corn or steam-flaked corn) were given to the animals individually in a milking stall barn during milking. Alfalfa hay was offered first; after all of the alfalfa hay was eaten (15 min later), corn silage was offered. The corn silage remaining (corn silage refusal) from the previous day was collected and weighed daily before morning feeding. The daily roughage intake per animal was calculated by subtracting the feed refusal from the feed allocated and then dividing the consumption per day by the number of animals in a group. The daily grain intake of the ewes was recorded individually in a milking stall barn twice a day. The ewes' dry matter (DM), crude protein (CP), and metabolizable energy (ME) intake values were calculated from the daily feed intake and the results of the chemical analyses of feeds that were used in the experiment. As the roughage sources were given in a group setting in the study, the data obtained from the ewes' roughage intake were not subjected to statistical analysis, and the groups' roughage intake results are presented as the mean ± the standard deviation. In the experiment, the ewes' corn grain and steam-flaked corn intake values were monitored individually, and these data were subjected to statistical analyses.

### 2.2 Feeds chemical analysis and in vitro gas production

Feed samples were collected at 15 d intervals during the study, oven-dried at 50 °C (Memmert GmbH + Co. KG, Germany), and ground in a mill with a sieve diameter of 1 mm (model HD-703, Şimşek Laborteknik Ltd., Türkiye). The feed DM content (method 930.15), CP (method 990.03), ether extract value (EE; method 920.39), and ash content (method, 942.05) were determined according to AOAC (2000). The neutral detergent fibre (NDF), acid detergent fibre (ADF), and acid detergent lignin (ADL) contents of the feed were analysed according to Van Soest et al. (1991) using an ANKOM 200 Fiber Analyzer (ANKOM Technology, Macedon, USA). The starch content of grains was determined according to the enzymatic–colorimetric method of Hall (2015). The daily rations given to the ewes and the ME contents of feeds were adopted from NRC (2007) (Table 1).

**Table 1:** Ewes' daily ration (kg d^−1^) and the chemical composition of the ingredients.

Groups	Feed stuff			
	Alfalfa hay	Corn silage	Corn grain	Steam-flaked corn
C	0.5	2.2	–	–
WC	0.4	1.7	0.6	–
FC	0.4	1.7	–	0.6
Item	Chemical composition			
DM (%)	90.48	30.42	90.25	90.12
CP (% DM)	20.90	8.59	8.59	8.88
EE (% DM)	1.90	1.83	3.56	2.83
NDF (% DM)	45.43	55.79	10.39	10.36
ADF (% DM)	33.87	40.90	4.68	5.48
ADL (% DM)	10.59	4.64	1.58	1.65
Ash (% DM)	10.72	3.83	2.68	1.73
Starch (% DM)	–	30.13	70.61	69.92
ME (Mcal ME per kilogram of DM)	2.1	2.6	3.2	3.4

The abbreviations used in the table are as follows: C – control; WC – whole corn; FC – steam-flaked corn; DM – dry matter; CP – crude protein; EE – ether extract; NDF – neutral detergent fibre; ADF – acid detergent fibre; ADL – acid detergent lignin; ME – metabolizable energy.

The in vitro gas production of whole and steam-flaked corn was also determined in the study. Fresh rumen inoculum was collected from three 7- to 8-month-old male goat kids at a local slaughterhouse (Pomet Company, Çanakkale, Türkiye). The rumen fluid samples were mixed in equal volumes, filtered through three-layered cheesecloth, and mixed with buffer solution; they were then placed on a magnetic stirrer at 39 °C under CO_2_ flux. Triplicate 200 ± 10 mg whole and steam-flaked corn samples were weighed into calibrated glass syringes (Fortuna, Germany), mixed with rumen fluid buffer (30 mL) solution ( 1:2 v/v ), and then incubated in a 39 °C water bath (Menke and Steingass, 1988). Gas production was recorded at 0, 3, 6, 9, 12, 24, and 48 h of incubation. Cumulative gas production data were fitted to the exponential equation reported by Orskov and McDonald (1979). ME and organic matter digestibility (OMD) of the whole corn and steam-flaked corn were calculated from the gas production according to Menke et al. (1979), as follows:

$$\begin{array}{c} \mathrm{ME}\;(\mathrm{MJ}\;\text{per kg DM})=2.20+0.136\;\mathrm{GP}+0.057\;\mathrm{CP},\\ \mathrm{OMD}\;(\%)=14.88+0.889\;\mathrm{GP}+0.45\;\mathrm{CP}+0.0651A, \end{array}$$

where GP is the 24 h gas production (mL), CP is the crude protein content (%), and A is the ash content (%).

### 2.3 Body weight (BW), body condition score (BCS), and milk yield

The ewes' BW was measured before the morning meal at 15 d intervals using an electronic bascule ( ± 20 g). BCS values were recorded at 15 d intervals by hand palpation of the lumbar region using a scale of 1 to 5 (where 1 denotes thin and 5 denotes obese) according to Russel (1984). BCS measurements were made by the same person in each measurement period. The milk yield of individual ewes was measured at 15 d intervals (at 08:30 and 16:30 LT) using a milking machine. During the experiment, milk yield measurement continued until the milk yield decreased by 50 mL d^−1^, and the milk yield measurement was carried out three times throughout the experiment. Milk samples were taken individually from the ewes during morning and evening milking (a total of 50 mL) to determine the fat-free solid content, fat content, protein content, and lactose content; this analysis was undertaken with an ultrasonic milk analyser (Milk-Lab Minor^®^, UK Ltd.).

### 2.4 Blood samplings and biochemical analysis

Blood samples of individual ewes were taken from the beginning of the experiment (Day 0), before ram introduction to ewes (at Day 15), and at the end of the study (Day 45). Blood samples were taken from the jugular vein using a Vacutainer tube (Becton and Dickinson, USA) 2 h after the morning meal. The blood samples were centrifuged for 10 min at 3500 rpm to separate the serum samples and then stored at - 20 °C until analysis. Serum glucose, urea, and cholesterol levels were analysed using a spectrophotometer (Shimadzu UV-1200, Kyoto, Japan) with commercial clinical chemistry test kits (ImproGen Diagnostic, Istanbul, Türkiye).

### 2.5 Oestrus detection and mating

In the animal production unit, rams were kept isolated from the ewes. As the “ram effect” was used to induce oestrus synchronization in this work, rams were brought close to the ewes' barns at the beginning of the experiment. For this purpose five Tahirova rams aged between 1 and 5 years were used for oestrus detection and mating. The rams were treated with REGULIN implants containing 54 mg melatonin (three pellets of REGULIN implants) that were placed subcutaneously near the ear a total of 45 d before the experiment. Oestrus detection was carried out twice a day (08:00 and 18:00 LT) using aproned rams. Ewes in oestrus were mated using the hand-mating method. If oestrus was observed in the morning, ewes were mated in the afternoon; if oestrus was detected in the afternoon, ewes were mated the next morning. Conception was determined as the time of the last oestrus and was finally confirmed with lambing. In the experiment, fertility was measured using the duration from ram introduction to the first oestrus (DRIE), the duration from ram introduction to conception (DRIC), the duration from ram introduction to lambing (DRIL), the non-return rate, the rate of mated ewes giving birth (lambing rate), and the lambs born per ewe lambing (litter size).

### 2.6 Statistical analyses

The in vitro gas production, OMD, and ME content of whole corn and steam-flaked corn were analysed by variance analysis. Ewes' BW values, BCS values, grain intake, serum analysis, milk yield, and milk composition data were analysed using repeated-measures variance analyses with the following statistical model:

1 $$y_{ijkl}=\mu +\alpha_{i\;}+\beta_{j}+E_{ik}+e_{ijkl}.$$

Here, $y_{ijkl}$ is an observed variable, μ is the general mean, $\alpha_{i}$ is the fixed group effect ( i= C, WC, or FC), $\beta_{j}$ is the fixed effect of observation day ( j= 1, …, 3; for grain intake j=1 , …, 6), $E_{ik}$ is the random repeated effect ( k=1 , …, 48), and $e_{ijkl}$ is the random error term. A Tukey test was used in the post hoc analysis.

Data on the DRIE, DRIC, DRIL, birth weight, and litter weight were analysed using the same procedure with the following statistical model:

2 $$y_{ijkl}=\mu +\alpha_{i}+\beta_{j}+E_{ik}+e_{ijkl}.$$

Here, $y_{ijkl}$ is an observed variable, μ is the general mean, $\alpha_{i}$ is the fixed group effect ( i= C, WC, or FC), $\beta_{j}$ is the fixed effect of ewe age ( j= 1, …, 4), $E_{ik}$ is a random repeated effect ( k=1 , …, 48), and $e_{ijk}$ is the random error term. A Tukey test was used in the post hoc analysis.

The litter size was analysed using the generalized estimating equation method (SAS, 1999) with the following statistical model:

3 $$y_{ijkl}=\Theta \left(\alpha_{i}++\Gamma_{j}+\Delta_{jk}\right).$$

Here, $y_{ijklm}$ is an observed variable, Θ is a function of the standard normal distribution, $\alpha_{i}$ is the fixed group effect ( i= C, WC, or FC), $\Gamma_{j}$ is the fixed effect of maternal age ( k=1 , …, 4), and $\Delta_{jk}$ is the random repeated effect ( l=1 , …, 48). A Wald chi-square test was used in the post hoc analysis. All analyses were performed with SAS (2004).

## 3 Results

### 3.1 In vitro gas production of corn grain and steam-flaked corn

**Figure 1:**
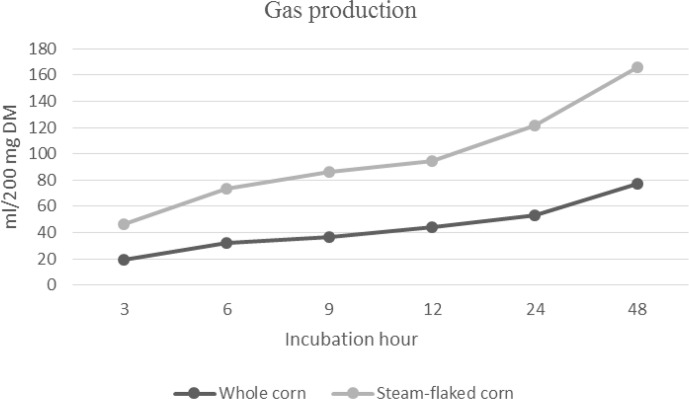
In vitro gas production of corn grain and steam-flaked corn.

The cumulative gas production of corn grain and steam-flaked corn is presented in Fig. 1. During the 96 h incubation period, the cumulative gas production values for corn grain and steam-flaked corn were determined to be 78.00 mL and 88.75 mL, respectively ( P=0.1144 ). The ME ( P=0.0122 ) and OMD ( P=0.0158 ) values of corn grain and steam–flaked corn were 14.34–16.52 MJ per kilogram of DM and 66.26 %–79.49 %, respectively. The ME level and OMD of grain corn were found to be lower compared with steam-flaked corn.

### 3.2 Feed intake, BW, and BCS

**Figure 2:**
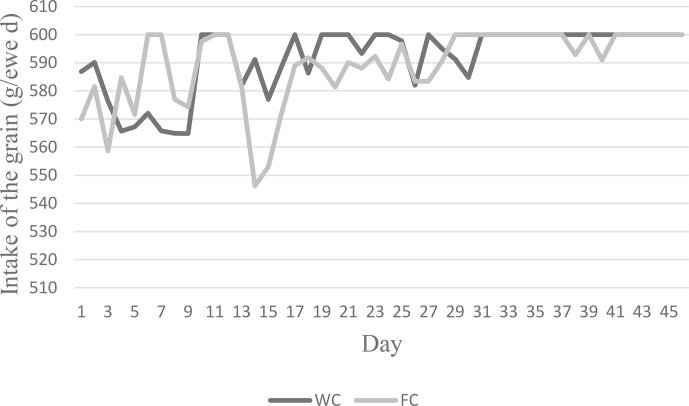
Changes in ewes' daily grain intake in the WC and FC groups.

The ewes' daily roughage dry matter intake (DMI) values varied between 0.90 and 1.16 kg d^−1^, whereas their CP intake varied between 115.83 and 147.82 g per kilogram of DM per day. The animals' daily roughage ME intakes in the C group were higher (2.78 Mcal ME) than in the WC and FC groups (Table 2). The daily change in the grain feed intake in the WC and FC groups is presented in Fig. 2.

**Table 2:** The mean ± the standard deviation of the daily roughage nutrient intake for the three experimental groups.

Parameters	C	WC	FC
DMI	1.16 ± 0.006	0.91 ± 0.002	0.90 ± 0.004
CPI	147.82 ± 0.269	116.70 ± 0.162	115.83 ± 0.191
MEI	2.78 ± 0.012	2.18 ± 0.018	2.13 ± 0.006

The abbreviations used in the table are as follows: C – control; WC – whole corn; FC – steam-flaked corn; DMI – dry matter intake (kg d^−1^); CPI – crude protein intake (grams per kilogram of DM); MEI – metabolizable energy intake (Mcal ME per kilogram of DM).

The DM, ME, and starch intake from grain feed were different among the experimental groups ( P<0.0001 ). The WC group had a higher, DM, ME, and starch intake than the FC group (Table 3).

**Table 3:** The least-squares mean ± the standard error of the mean and the P values for the daily grain nutrient intake for the three experimental groups.

Parameters	WC	FC	P value
DMI	0.54 ± 0.002	0.42 ± 0.002	<0.0001
MEI	1.73 ± 0.002	1.43 ± 0.005	<0.0001
SI	383.94 ± 1.218	290.25 ± 1.22	<0.0001

The abbreviations used in the table are as follows: WC – whole corn; FC – steam-flaked corn; DMI – dry matter intake (kg); MEI – metabolizable energy intake (Mcal ME per kilogram of DM); SI – starch intake (grams per kilogram of DM).

**Figure 3:**
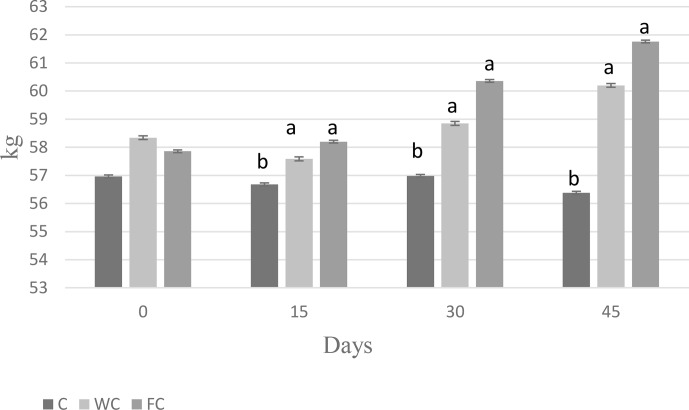
Changes in body weight among the three experimental groups. Letters indicate significant differences between groups ( P≤0.05 ).

**Figure 4:**
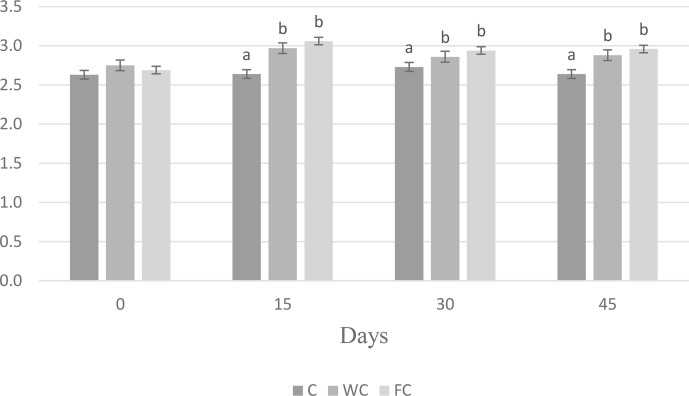
Changes in the BCS among the three experimental groups. Letters indicate significant differences between groups ( P≤0.05 ).

There were no differences in the BW ( P=0.8116 ) or BCS ( P=0.7696 ) among the groups at the beginning of the experiment (Day 0). The BW of the C group was lower than that of the WC and FC groups ( P=0.0031 ). The BW (P=0.0031 ) and BCS ( P<0.0001 ) values of the WC and FC groups were significantly higher than those of the C group (Table 4). The BW was affected by the measurement day ( P=0.0378 ) but was not affected by the group × measurement day interaction ( P=0.4306 ). On the contrary, the BCS was not affected by the measurement day ( P=0.1559 ) but was affected by the group × measurement day interaction ( P=0.0335 ). Ewes in the C group lost 0.58 kg of BW, whereas ewes in the WC and FC groups gained 1.35 and 3.9 kg, respectively, during the experiment (Fig. 3). The change in the BCS is shown in Fig. 4. As seen in Fig. 4, the BCS of ewes in the C group did not change during the experiment, whereas the BCS of ewes in the WC and FC groups increased by 0.13 and 0.27 points, respectively.

**Table 4:** The least-squares mean ± the standard error of the mean and the P values for the BW and BCS values of the three experimental groups.

Groups	C	WC	FC	P value
BW	56.58^b^ ± 0.729	58.82^a^ ± 0.735	60.10^a^ ± 0.720	0.0031
BCS	2.64 ^b^ ± 0.036	2.89^a^ ± 0.036	2.96^a^ ± 0.036	<0.0001

^a,b^ Means with different superscripts in the same row are significantly different ( P≤0.05 ). The abbreviations used in the table are as follows: C – control; WC – whole corn; FC – steam-flaked corn; BW – body weight (kg); BCS – body condition score.

### 3.3 Milk yield and composition

**Figure 5:**
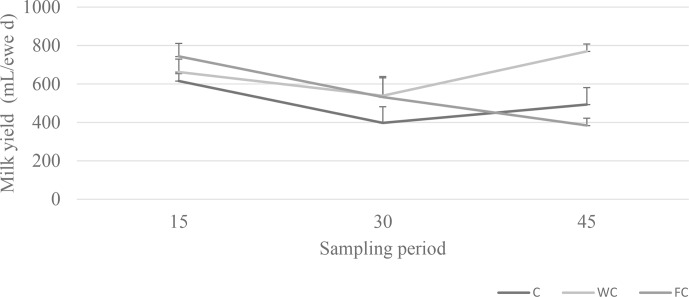
Changes in the milk yield among the three experimental groups.

Milk yield was not affected by the nutritional treatments or the group × sampling period interaction ( P>0.05 ), but it was affected by the sampling period ( P=0.0009 ; Fig. 5). The milk yield of ewes in the FC group decreased as the experiment progressed.

The milk composition (fat, non-fat solid, protein, and lactose) changed significantly as a function of the sampling period ( P<0.0001 ). However, the milk nutrient composition was not affected by the group × sampling period interaction ( P>0.05 ). The milk fat and non-fat solid content values of ewes in the WC group were significantly lower than those of ewes in the C and FC groups (Table 5). The milk protein ( P=0.1532 ) and lactose content values were found to be similar in all groups ( P=0.2657 ).

**Table 5:** The least-squares mean ± the standard error of the mean and the P values for the milk nutrient composition (%) of the three experimental groups.

Item	C	WC	FC	P value
Fat	9.49^a^ ± 0.597	7.71^b^ ± 0.324	8.13^a^ ± 0.438	0.0375
Non-fat solid	9.99^a^ ± 0.240	9.45^b^ ± 0.131	9.90^a^ ± 0.176	0.0468
Protein	4.72 ± 0.157	4.47 ± 0.086	4.63 ± 0.116	0.1532
Lactose	4.50 ± 0.117	4.26 ± 0.064	4.48 ± 0.086	0.2657

^a,b^ Means with different superscripts in the same row are significantly different ( P≤0.05 ). The abbreviations used in the table are as follows: C – control; WC – whole corn; FC – steam-flaked corn.

### 3.4 Serum parameters

Serum glucose ( P=0.6786 ) and cholesterol ( P=0.4244 ) concentrations were found to be similar in the groups (Table 6). However, the serum urea concentrations differed significantly among the groups ( P=0.0450 ). It was determined that the C group had a significantly lower serum urea concentration compared with the other groups ( P≤0.05 ).

**Table 6:** The least-squares mean ± the standard error of the mean of the serum metabolites in the three experimental groups (in mg dL^−1^).

Serum parameters	Groups			P value
	C	WC	FC	
Glucose	44.70 ± 0.809	43.32 ± 0.806	42.64 ± 0.808	0.6786
Urea	8.76^b^ ± 0.086	9.09^a^ ± 0.086	8.95^a^ ± 0.086	0.0450
Cholesterol	59.98 ± 1.922	60.56 ± 1.833	57.36 ± 1.833	0.4244

^a,b^ Means with different superscripts in the same row are significantly different ( P≤0.05 ). The abbreviations used in the table are as follows: C – control; WC – whole corn; FC – steam-flaked corn.

**Figure 6:**
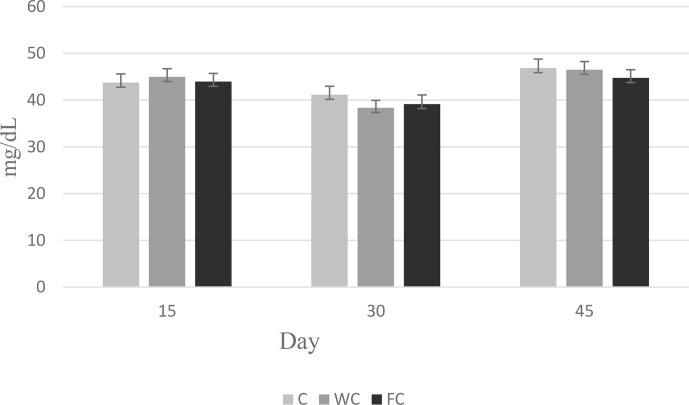
Change in the serum glucose concentration in the three experimental groups according to the sampling period.

**Figure 7:**
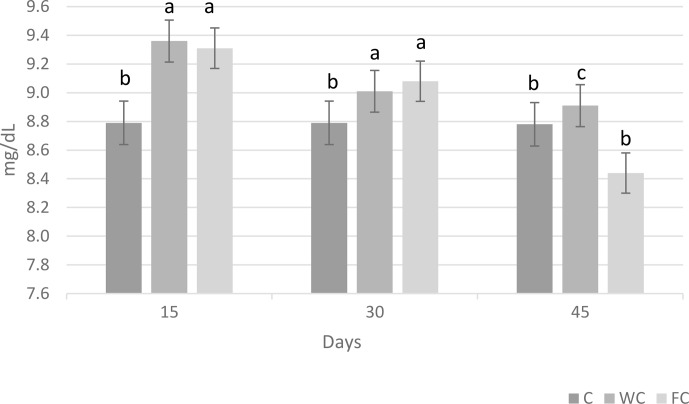
Change in the serum urea concentration in the three experimental groups according to the sampling period. Letters indicate significant differences between sampling periods ( P≤0.05 ).

**Figure 8:**
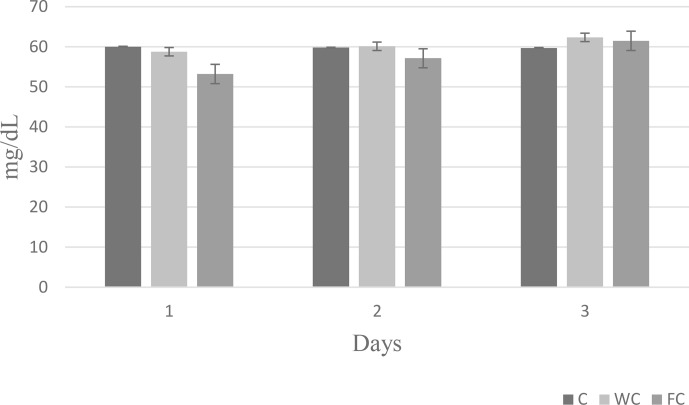
Change in the serum cholesterol concentration in the three experimental groups according to the sampling period.

The changes in the blood glucose, urea, and cholesterol concentrations of the ewes are presented in Figs. 6, 7, and 8, respectively. The serum glucose ( P<0.0001 ) and urea ( P=0.0021 ) concentration were significantly affected by the sampling period. The serum parameters were not affected by the group × sampling period interaction, age, sampling period × age interaction, group × age interaction, or group × sampling period × age interaction ( P>0.05 ).

### 3.5 Reproductive performance

The DRIE ( P = 0.5313), DRIC ( P = 0.7413), DRIL ( P = 0.3936), birth weight ( P = 0.6746), and litter weight ( P = 0.5120) were found to be similar among the experimental groups (Table 7). Although the non-return ratio ( P = 0.0996) and litter size ( P = 0.1136) were found to be similar in the groups, the lambing ratio of the FC group was higher than the C group ( P = 0.0311).

**Table 7:** Mean ± the standard error of the mean of the reproductive performance of the three experimental groups.

Traits	C	WC	FC	P value
DRIE (d)	10.1 ± 2.28	12.3 ± 1.73	10.6 ± 1.99	0.5313
DRIC (d)	12.6 ± 2.44	13.3 ± 1.95	10.6 ± 1.99	0.7413
DRIL (d)	149.4 ± 2.27	151.3 ± 4.33	146.0 ± 0.46	0.3936
Non-return rate (%)	81.2 ± 10.07	93.7 ± 6.25	100 ± 0.0	0.0996
Lambing rate (%)	75.0^b^ ± 11.18b	93.7^ab^ ± 6.25ab	100.0^a^ ± 0.00	0.0311
Litter size	1.42 ± 0.13	1.73 ± 0.15	1.62 ± 0.12	0.1136
Birth weight (kg)	4.37 ± 0.25	4.26 ± 0.19	4.54 ± 0.21	0.6746
Litter weight (kg)	6.38 ± 0.61	7.39 ± 0.53	7.38 ± 0.55	0.5120

^a,b^ Means with different superscripts in the same row are significantly different ( P≤0.05 ). The abbreviations used in the table are as follows: C – control; WC – whole corn; FC – steam-flaked corn; DRIE – duration from ram introduction to the first oestrus; DRIC – duration from ram introduction to conception; DRIL – duration from ram introduction to lambing.

## 4 Discussion

The ruminal starch degradability of corn grain can be altered by the processing technique, such as cracking, grinding, or steam-flaking (Firkins et al., 2001). The steam-flaking process increases the degradability of starch from grains in the rumen (Huntington, 1997), as observed in this study. It has been reported that the ruminal digestibility of corn is 61.5 % when the grain is given whole, 78.3 % when it is ground, and 91.3 % when it is extruded (Landau et al., 1992). Huntington (1997) reported that the ruminal digestibility of steam-flaked corn was 84.8 %.

The respective OMD values of corn grain and steam-flaked corn were found to be 66.26 % and 79.49 % in our study. The OMD value in this work was similar to that reported by Landau et al. (1992) but lower than that reported by Karami et al. (2018), who reported that the OMD values of corn grain and steam-flaked corn were 83.15 % and 80.41 %, respectively. The difference in digestibility value between corn grain and steam-flaked corn found in different studies could be explained by the physical and chemical properties of grain cultivars (Yang et al., 2014), along with growing conditions and agronomic properties (Dehghan-banadaky et al., 2007; Nikkhah, 2012).

The DMI from whole corn was higher than that from steam-flaked corn in this study. Steam-flaked corn supplementation decreased the DMI by 0.12 kg d^−1^ compared with the WC group (0.42 vs. 0.54 kg d^−1^). This reduction in the DMI could be attributed to the greater ruminal fermentation of steam-flaked corn; this could lead to an increase in propionate production in the rumen, resulting in higher hypophagic effects, compared with acetate (Sheperd and Combs, 1998). It has been reported that the use of starch sources with high ruminal digestibility reduces the DMI in dairy cows (Yang et al., 2000; Oba and Allen, 2003). Although the WC group consumed more starch and energy than the FC group, there were no differences between the WC and FC groups in terms of the BW and BCS; in contrast, the C group had a significantly lower BW and BCS than the other groups. Scaramuzzi et al. (2006) reported that feed flushing positively affects the BW and BCS of ewes. Similarly, it has been reported that the supplementation of concentrate at a level of 1.5 % of the animal's BW in ewes significantly increased BW gain (Naqvi et al., 2013). Cirne et al. (2016), who evaluated the reproductive performance of Île-de-France ewes under grazing conditions, stated that 0.5 kg d^−1^ concentrate supplementation significantly increased animals' BW and BCS.

In this study, the milk yield of ewes was not affected by whole or steam-flaked corn supplementation, whereas the milk fat and non-fat solid content values were significantly decreased by whole-corn supplementation. The reason for the reduction in milk fat and non-fat solids observed in the WC group could be that they consumed more grain feed than the FC group, while the C group had a daily feed intake that was primarily made up of coarse forage sources. Ewes' milk quality has been determined to be affected by the ration forage : concentrate ratio in several studies (Mele et al., 2006; Sanz-Sampleyo et al., 2007; Martini et al., 2010). An increase in the forage : concentrate ratio causes an increase in acetate production by cellulolytic bacteria in the rumen. Additionally, the mammary gland uses acetate as a precursor for fat synthesis, leading to an increase in the milk fat content (Chesson and Forsberg, 1997; Buccioni et al., 2012).

Whole and steam-flaked corn supplementation did not affect the serum glucose concentration in the experimental groups. In contrast, supplementation with corn grain is expected to increase the duodenal flow of glucose and increase the blood glucose concentration (Landau et al., 1992; Banchero et al., 2007). Blood glucose concentrations do not usually increase when corn is fed to ruminants. In goats, supplementation with corn at 1.5 times the maintenance requirements vs. 1.0 times the maintenance requirements did not increase the blood glucose concentration (Nougeria et al., 2017). In another study, goats supplemented with 2.5 times their maintenance requirements showed an increased blood glucose concentration (Haruna et al., 2009). Thus, the energy levels fed to the ewes used in our study were probably not sufficient to enhance the serum glucose concentration. In addition, the blood samples were collected 2 h after the morning feeding in our study. Due to the post-feed intake rumen volume and weight increase, this would activate neuroendocrine pathways and, in turn, may lead to the stimulation of insulin secretion (Zhang et al., 2004). Plasma insulin concentrations were found to increase 2 h after feeding in ewes, and the glucose concentration decreased accordingly (Marie et al., 2001). Therefore, an increase in the serum glucose concentration may not have been observed. In this study, the serum urea concentration was higher in ewes in the WC and FC groups than in the C group. Ruminal nitrogen losses usually increase due to an imbalance between the level of degradable nitrogen and fermentable carbohydrates in the feed ration. This leads to nitrogen in the feed not being used effectively in the rumen, an increase in the absorption of ammonia nitrogen from the rumen wall, and an increase in the concentration of urea in the blood (Peyraud and Delagarde, 2013). This situation can be alleviated by using feed sources that contain carbohydrates that have high intraruminal degradability (grind corn, flake corn, etc.) (Rius et al., 2010). In this way, ammonia production can be reduced by providing energy to microorganisms in the rumen. However, contrary to reports in the literature, the serum urea concentrations were found to be high in both the FC and WC groups in the present study. Serum cholesterol concentration is an indicator of energy metabolism in the body. It has been reported that there is an increase in the serum cholesterol levels in ewes fed a high-energy ration compared with those not fed a high-energy ration (Senosy et al., 2013). Cholesterol can affect the reproductive performance of ruminants, as it is a precursor of hormones that affect reproduction. No difference was determined in terms of the serum cholesterol concentrations in the present study. The serum glucose (28.70–61.2 mg dL^−1^), urea (8–23.9 mg dL^−1^), and cholesterol (45–75.7 mg dL^−1^) concentrations observed in the study were within the reported range for ewes (Macías-Cruz et al., 2017; Yıldırır et al., 2022). Many studies in the literature have reported that ovulation rates are enhanced by increasing the energy level of the diet (Downing et al., 1995; Letelier et al., 2008; Banchero et al., 2020). De Santiago-Miramontes et al. (2008) reported that supplementary feeding with 290 g corn and 140 g soybean significantly increased the ovulation rate compared with non-supplemented feeding in goats. In addition, the ovulation rate was increased when corn was added to the diet and provided at 1.5 times the maintenance metabolizable energy in goats (Nougeria et al., 2017). Letelier et al. (2008) suggested that supplementing steam-flaked corn increases the ovulation rate in ewes. In our study, corn and steam–flaked corn were used to determine the effects of the starch degradability levels in the rumen on the reproductive performance of ewes. It is reported that corn starch is partly degradable in the rumen. Past studies have found that the supplementation of corn increases the glucose supply to the small intestine and leads to an increased ovulation rate (Landau et al., 1997; Banchero et al., 2007). In contrast, in the current study, corn supplementation did not increase serum glucose concentration, but the numerically highest litter size was determined in the WC group (1.73), followed by the FC (1.62) and C (1.42) groups. In profitable ewe production, it is desirable to obtain more lambs from each ewe. The numerical difference observed in the rate of multiple births in the study is not statistically significant, but it is important from a practical point of view. It is thought that sample size may have an impact on this situation. In this study, the highest lambing rate was observed in the FC group (100 %), followed by the WC (93.7 %) and C (75 %) groups. It has been reported that the rate of conception in ewes normally ranges from 80 % to 90 % in well-managed herds during the breeding season (Davies, 2019). The lambing rate of the C group was below the lower limit reported by Davies (2019), whereas this value was above the upper limit in both the WC and FC groups. On the other hand, according to Tesfaye et al. (2023), the BCS of the ewes that were supplemented exceeded 3, resulting in economic benefits via an increased lambing rate. Similarly, the improvement in the ewes' BCS significantly increased the lambing rate, whereas the lambing rate significantly decreased for BCS values below 2.5 (El-Shahat and Abdel, 2011). In this study, which is in line with literature reports, it is thought that supplementation with whole corn and steamed corn increased the BCS to approximately 3 and that this may have been effective with respect to increasing the lambing rate in the experimental groups. It had also been reported that the BCS may affect the return rate and that a ewe with an average BCS of 2.5–4 has a lower return rate (Cam et al., 2018). The average non-return rates for groups in this study were similar. In addition, for ewes, it has been reported that feed flushing results in an increasing trend with respect to the BCS and BW and that this effect can persist throughout the lambing period. Additionally, lambs born from feed-flushed ewes had higher birth weights (Tesfaye et al., 2023). In a recent study, it was observed that feed flushing had no impact on either birth weight or litter weight, which is in agreement with the finding of Makela et al. (2022).

A total of 17–18 d is necessary for intense oestrus behaviour in ewes, as is commonly known (Rosa and Bryant, 2002; Maldonado et al., 2021). Dursun (2022) reported that estrus behaviours appeared 12 d after ram introduction. These values are similar to the values obtained from this study: a change was noted between 10 and 12 d after ram introduction. The duration from ram introduction to lambing includes conception, implantation, and embryonic losses – in short, the total pregnancy process. However, there was no difference between the DRIC and the DRIL in this study. Flushing feeding is reported to increase the rate of conception during the breeding season (Lassoued et al., 2009; Tesfaye et al., 2023). Differences in findings across studies may be due to differences in feed flushing, such as the amount or type of supplementary feed and the duration of feeding.

## 5 Conclusions

Feed flushing with whole corn and steam-flaked corn for 15 d before ram introduction and for 30 d during mating increased the BW, BCS, and lambing rate in ewes. The results of this study show that whole corn and flake corn are important sources of energy for ewes. When these feeds are accessible and economical (cheap), they are important energy sources for sheep and increase the litter size and lambing rate. In subsequent studies, the effects of shorter-term supplementary feeding using different energy sources on reproductive performance and hormonal profiles need to be evaluated.

## Data Availability

Data are available from the authors upon reasonable request.

## References

[bib1.bib1] AOAC (2000). Official Methods of Analysis.

[bib1.bib2] Banchero GE, Quintans G, Vazquez A, Gigena F, Manna ALa, Lindsay DR, Milton JBT (2007). Effect of supplementation of ewes with barley or corn during the last week of pregnancy on colostrum production. Animal.

[bib1.bib3] Banchero GE, Stefanova K, Lindsay DR, Quintans G, Baldi F, Milton JTB, Martin GB (2020). Ovulation and ovulation rate in ewes under grazing conditions: factors affecting the response to short-term supplementation. Animal.

[bib1.bib4] Boland MP, Lonergan P, O'Callaghan D (2001). Effect of nutrition on endocrine parameters, ovarian physiology, and oocyte and embryo development. Theriogenology.

[bib1.bib5] Buccioni A, Decandia M, Minieri S, Molle G, Cabiddu A (2012). Lipid metabolism in the rumen: new insights on lipolysis and biohydrogenation with an emphasis on the role of endogenous plant factors. Anim Feed Sci Tech.

[bib1.bib6] Cam MA, Garipoglu AV, Kirikci K (2018). Body condition status at mating affects gestation length, offspring yield and return rate in ewes. Arch Anim Breed.

[bib1.bib7] Chesson A, Forsberg CW, Hobson PN, Stewart CS (1997). The rumen microbial ecosystem.

[bib1.bib8] Cirne LGA, Da Silva SAG, Olivera MEF, Barbosa JC, Carneiro de OGJ, Bagaldo AR, Pinto de Carvalho GG, Moreno GMB (2016). Reproductive performance of Ile de France ewes under dietary supplementation before and during the breeding season. Semina Ciências Agrárias.

[bib1.bib9] Macías-Cruz U, Vicente-Pérez R, Correa-Calderón A, Mellado M, Meza-Herrera CAL, Avendaño-Reyes L (2017). Undernutrition pre- and post-mating affects serum levels of glucose, cholesterol and progesterone, but not the reproductive efficiency of crossbred hair ewes synchronized for estrus. Livest Sci.

[bib1.bib10] Davies P (2019). Veterinary Reproduction and Obstetrics.

[bib1.bib11] De Santiago-Miramontes MA, Rivas-Munoz R, Munoz-Gutierrez M, Malpoux B, Scaramuzzi RJ, Delgadillo JA (2008). The ovulation rate in anooestrus female goats managed under grazing conditions and exposed to male effects is increased by nutritional supplementation. Anim Reprod Sci.

[bib1.bib12] Dehghan-banadaky M, Corbett R, Oba M (2007). Effects of barley grain processing on productivity of cattle. Anim Feed Sci Technol.

[bib1.bib13] Ding Y, Liu X, Guan Y, Li Z, Luo M, Wu D, Ye L, Guo L, Wang L, Guan Y (2024). Balancing nutrition for successful in ruminants. Modern Agricul.

[bib1.bib14] Downing JA, Joss J, Connell P, Scaramuzzi RJ (1995). Ovulation rate and the concentration of gonadotrophic and metabolic hormones in ewes fed lupin grain. J Reprod Fertil.

[bib1.bib15] Dursun Ş (2022). The effect of different ram introduction procedures (protocol 66) on fertility parameters in Anatolian Merino ewes during breeding season. Turk J Vet Anim Sci.

[bib1.bib16] El-Shahat K, Abdel MU (2011). Effects of dietary supplementation with vitamin E and selenium on metabolic and reproductive performance of Egyptian Baladi ewes under subtropical conditions. World Appl Sci J.

[bib1.bib17] Frinks JL, Eastridge ML, St-Pierre NR, Noftsger SM (2001). Effects of grain variability and processing on starch utilization by lactating dairy cattle. J Anim Sci.

[bib1.bib18] Gomez LM, Posada SL, Olivera-Angel M (2016). Starch in ruminant diets: A review. Rev Colomb Cienc Pec.

[bib1.bib19] Habibizad J, Riasi A, Kohram H, Rahmani HR (2015). Effect of long-term or short-term supplementation of high energy or high energy-protein diets on ovarian follicles and blood metabolites and hormones in ewes. Small Ruminant Res.

[bib1.bib20] Hall MB (2015). Determination of dietary starch in animal feeds and pet food by an enzymatic-colorimetric method: Collaborative study. J AOAC Int.

[bib1.bib21] Haruna S, Kuroiwa T, Wengeng LU, Zabuli J, Tanaka T, Kamomae H (2009). The Effects of short-term nutritional stimulus before and after the luteolysis on metabolic status, reproductive hormones and ovarian activity in goats. J Reprod Develop.

[bib1.bib22] Huntington GB (1997). Starch utilization by ruminants: from basics to the bunk. J Anim Sci.

[bib1.bib23] Karami M, Palizdar MH, Almazi MS (2018). The effect of different processing of corn grain on gas production kinetics and *in vitro* digestibility in Taleshi cows. J Livest Sci.

[bib1.bib24] Kebede HG (2019). Estimates of Genetic Parameters and Genetic Trends for Productive and Reproductive Traits of Doyogena Sheep in Southern Ethiopia [master's thesis].

[bib1.bib25] Koyuncu M, Canbolat O (2009). Effects of different dietary energy levels on the reproductive performance of Kivircik sheep under a semi-intensive systems in South Marmara Region on Turkey. J Anim Feed Sci.

[bib1.bib26] Landau S, Nitsan Z, Zoref Z, Madar Z (1992). The influence of processing corn grain on glucose metabolism in ewes. Reprod Nutr Dev.

[bib1.bib27] Landau S, Bor A, Leibovich H, Zoref Z, Nitsan Z, Madar Z (1995). The effects of ruminal starch degradability in the diet of Booroola crossbreed ewes on induced ovulation rate and prolificacy. Anim Reprod Sci.

[bib1.bib28] Lassoued N, Rekik M, Ben Salem H (2009). Influence of supplementary feeding and the ram effect on conception rate of Barbarine ewes during spring mating, Nutritional and foraging ecology of sheep and goats. Options Méditerranéennes Série A Séminaires Méditerranéens.

[bib1.bib29] Letelier C, Mallo F, Encinas T, Ros JM, Gonzalez-Bulnes A (2008). Glucogenic supply increase ovulation rate by modifying follicle recruitment and subsequent development of preovulatory follicles without effects on ghrelin secretion. Soc Reprod Fert.

[bib1.bib30] Makela B, Recktenwald E, Alves FC, Ehrhardt R, Lopez A (2022). Effects of pre-conceptional nutrition and season on fetal growth during pregnancy in sheep. Theriogenology.

[bib1.bib31] Maldonado JG, Valverde GR, Rodriguez De Lara R, Gallegos Sanchez J, Antillon RJ (2021). Ram sexual preferences and estrus behavior expression in ewes with different reproductive status. Turk J Vet Anim Sci.

[bib1.bib32] Marie M, Findlay PA, Thomas L, Adam CL (2001). Daily patterns of plasma leptin in sheep: effects of photoperiod and food intake. J Endocrinol.

[bib1.bib33] Martin GB, Blache D, Miller DW, Vercoe PE (2010). Interactions between nutrition and reproduction in the management of the male ruminant. Animal.

[bib1.bib34] Martini M, Liponi GB, Salari F (2010). Effect of forage: concentrate ratio on the quality of ewe's milk, especially on milk fat globules characteristics and fatty acids composition. J Dairy Res.

[bib1.bib35] Mele M, Buccioni A, Petacchi F, Serra A, Banni S, Antongiovanni M, Secchiari P (2006). Effect of forage/concentrate ratio and soybean oil supplementation on milk yield, and composition from Sarda ewes. Anim Res.

[bib1.bib36] Menke KH, Steingass H (1988). Estimation of the energetic feed value obtain from the chemical analysis and in-vitro gas production using rumen fluid. Anim Res Dev.

[bib1.bib37] Menke KH, Raab L, Salewski A, Steingas H, Fritz D, Schneider H (1979). The estimation of the digestibility and metabolizable energy content of ruminant feeding stuffs from the gas production when they are incubated with rumen liquor. J Agr Sci Cambr.

[bib1.bib38] Moran AW, Al-Rammahi M, Zhang C, Bravo D, Calsamiglia S, Shirazi-Beechey SP (2014). Sweet taste receptor expression in ruminant intestine and its activation by artificial sweeteners to regulate glucose absorption. J Dairy Sci.

[bib1.bib39] Naqvi M, Sejian V, Karim SA (2013). Effect of feed flushing during summer season on growth, reproductive performance and blood metabolites in Malpura ewes under semiarid tropical environment. Trop Anim Health Pro.

[bib1.bib40] Nikkhah A (2012). Barley grain for ruminants: A global treasure or tragedy. J Anim Sci Biotechnol.

[bib1.bib41] Nougeria DM, Eshtaeba A, Cavalieri J, Fitzpatrick LA, Gummow B, Blache D, Parker AJ (2017). Short-term supplementation with maize increases ovulation rate in goats when dietary metabolizable energy provides requirements for both maintenance and 1.5 times maintenance. Theriogenology.

[bib1.bib42] NRC (2007). Nutritional Research Council, Nutrient Requirements of Small Ruminants.

[bib1.bib43] Oba M, Allen MS (2003). Effects of corn grain conservation method on feeding behavior and productivity of lactating dairy cows at two dietary starch concentrations. J Dairy Sci.

[bib1.bib44] Orskov ER, McDonald I (1979). The estimation of protein degradability in the rumen from incubation measurements weighted according to rate of Passage. J Agr Sci Cambr.

[bib1.bib45] Palizdar MH, Mohammadian-Tabrizi HR, Pourelmi MR, Rafiee F (2014). Effect of using steam flaked and extruded corn grain in total mixed ration on *in vitro* rumen fermentation kinetics and gas production. Res Opin Anim Vet Sci.

[bib1.bib46] Peyraud JL, Delagarde R (2013). Managing variations in dairy cow nutrient supply under grazing. Animal.

[bib1.bib47] Philippeau C, Martin C, Michalet-Doreau B (1999). Influence of grain source on ruminal characteristics and rate, site, and extent of digestion in beef steers. J Anim Sci.

[bib1.bib48] Rigout S, Hurtaud C, Lemosquet S, Bach A, Rulquin H (2003). Lactational Effect of Propionic Acid and Duodenal Glucose in Cows. J Dairy Sci.

[bib1.bib49] Rius AG, McGiliard ML, Umberger CA, Hanigan MD (2010). Interactions of energy and predicted metabolizable protein in determining nitrogen efficiency in the lactating dairy cow. J Dairy Sci.

[bib1.bib50] Rosa HJD, Bryant MJ (2002). The “ran effect” as a way of modifying the reproductive activity in the ewe. Small Ruminant Res.

[bib1.bib51] Russel A (1984). Body condition scoring of sheep. In Pract.

[bib1.bib52] Sanz-Sampleyo MR, Chilliard Y, Schmidely P, Boza J (2007). Influence of type of diet on the fat constitutes of goat and sheep milk. Small Ruminant Res.

[bib1.bib53] SAS (2004). SAS OnlineDoc^®^, Version 9.0.

[bib1.bib54] Scaramuzzi R, Campbell B, Downing J, Kendall NR, Khalid M, Munoz-Gutierrez M, Somchit A (2006). A review of the effects of supplementary nutrition in the ewe on the concentrations of reproductive and metabolic hormones and the mechanisms that regulate folliculogenesis and ovulation rate. Reprod Nutr Dev.

[bib1.bib55] Senosy W, Abdel-Raheem ShM, Abd-Allah M, Famy S, Hassan EH, Derar RI (2013). Effect of transient high energy diets just after ovulation on ovarian performance and metabolic status in cyclic ewes. Small Ruminnat Res.

[bib1.bib56] Sheperd A, Combs D (1998). Long-term effects of acetate and propionate on voluntary feed intake by mid lactation cows. J Dairy Sci.

[bib1.bib57] Swan CG, Bowman JGP, Martin JM, Giroux MJ (2006). Increased puroindoline levels slow ruminal digestion of wheat (*Triticum aestivum L.*) starch by cattle. J Anim Sci.

[bib1.bib58] Tesfaye A, Asmare B, Abiso T, Wamatu J (2023). Effect of Nutritional Flushing Using Long Term Energy and Protein Supplementation on Growth Performance and Reproductive Parameters of Doyogena Ewes in Ethiopia. Vet Sci.

[bib1.bib59] Van Barneveld SL (1999). Chemical and physical characteristics of grains related to variability in energy and amino acid availability in ruminants: A review. Aust J Agr Res.

[bib1.bib60] Van Soest PJ, Robertson JB, Lewis BA (1991). Methods for dietary fiber, neutral detergent fiber, and nonstarch polysaccharides in relation to animal nutrition. J Dairy Sci.

[bib1.bib61] Wang Y, Jin C, Yi Y, Hu Y, Han X, Tan Z, Wang Z, Kang J (2023). Rumen-Protected Glucose Stimulates the Secretion of Reproductive Hormones and the mTOR/AKT Signaing Pathway in the Ovares of Early Postpartum. Sci Rep.

[bib1.bib62] Yang WZ, Beauchemin KA, Rode LM (2000). Effects of barley grain processing on extent of digestion and milk production of lactating cows. J Dairy Sci.

[bib1.bib63] Yang L, Chen J, Cheng X, Xi D, Yang S, Deng W, Mao H (2014). Phylogenetic Analysis of 16S rRNA Gene Sequences Reveals Rumen Bacterial Diversity in Yaks (Bos grunniens). Molecul Biol Rep.

[bib1.bib64] Yıldırır M, Çakır DÜ, Yurtman İY (2022). Effects of restricted nutrition and flushing on reproductive performance and metabolic profiles in sheep. Livest Sci.

[bib1.bib65] Zhang S, Blache D, Blackberry MA, Martin GB (2004). Dynamics of the responses in secretion of luteinizing hormone, leptin and insulin following an acute increase in nutrition in mature male sheep. Reprod Fert Develop.

